# Effects of exercise combined with whole body vibration in patients with patellofemoral pain syndrome: a randomised-controlled clinical trial

**DOI:** 10.1186/s12891-020-03599-2

**Published:** 2020-08-28

**Authors:** Angel Yañez-Álvarez, Beatriz Bermúdez-Pulgarín, Sergio Hernández-Sánchez, Manuel Albornoz-Cabello

**Affiliations:** 1 Department of Physiotherapy, Faculty of Nursing, Physiotherapy and Podiatry, University of Seville, 41009 Seville, Spain; 2 Department of Celular Biology, University of Seville, 41009 Seville, Spain; 3 Physiotherapy Area, Traslational Research Centre of Physiotherapy, Miguel Hernandez University, Sant Joan d’Alacant, 03550 Alicante, Spain

**Keywords:** Anterior knee pain, Whole body vibration, Therapeutic exercise

## Abstract

**Background:**

Patellofemoral pain is a prevalent condition in the general population, especially in women, and produces functional impairment in patients. Therapeutic exercise is considered an essential part of the conservative management. The use of vibration platforms may help improve strength and function and reduce pain in patients with knee disorders. The aim of this investigation was to determine the effects of adding whole body vibration (vertical, vibration frequency of 40 Hz, with an amplitude from 2 to 4 mm) to an exercise protocol for pain and disability in adults with patellofemoral pain.

**Methods:**

A randomised clinical trial was designed, where 50 subjects were randomly distributed into either an exercise group plus whole body vibration or a control group. Pain, knee function (self-reported questionnaire) and range of motion and lower limb functionality were assessed at baseline and at 4 weeks. The experimental group performed 12 supervised sessions of hip, knee and core strengthening exercises on a vibration platform 3 times per week during 4 weeks. The control group followed the same protocol but without vibration stimuli. Differences in outcome measures were explored using an analysis of the variance of 2 repeated measures. Effect sizes were estimated using Square Eta (η^2^). Significant level was set al *P* < 0.05.

**Results:**

Statistically significant differences were found after intervention in favour of the experimental group in the between-groups comparison and in the interaction of the experimental group before and after treatment in terms of pain perception (*P* = 0.000; η^2^ = 0.63) and function outcomes scores (P = 0.000; η^2^ 0.39 and 0.51 for lower limb functional scale and Kujala scores respectively).

**Conclusion:**

A 4-week whole body vibration exercise programme reduces pain level intensity and improves lower limb functionality in patellofemoral pain patients and is more effective than exercise alone in improving pain and function in the short-term.

**Trial registration:**

ClinicalTrials.gov (NCT04031248). This study was prospectively registered on the 24th July, 2019.

## Background

Knee pain is a common condition with a high prevalence in the general population, with patellofemoral pain (PFP) being one of the most common forms of knee pain [1]. Patellofemoral pain is described as a diffuse non-traumatic pain in the anterior area of the knee, which is aggravated when performing patellofemoral joint loading activities such as squats, running, going up and down stairs or sitting for a long time [2]. Its annual prevalence is approximately 23% in adults, increasing to 29% in adolescents; and it is more frequent in women [3].

There is no specific cause of pain in these patients and the aetiology of PFP is considered multifactorial. For this reason, the diagnosis is complex and essentially based on the clinical history, always ruling out lesions of adjacent structures such as ligaments or menisci [2]. This syndrome is considered to be a ‘black hole’ in orthopaedic medicine because no single explanation clarifies the patellofemoral problem and no single therapeutic intervention can reduce all patellofemoral dysfunction [4].

Pain around or behind the patella, weakened knee extensor muscles and associated symptoms usually reduce knee functionality and entail a limitation in daily and occupational activities, negatively impacting a patient’s quality of life and social aspects [5].

Exercise therapy is an evidenced-based non-pharmacological intervention often prescribed for this condition [6]. There is strong evidence that supports its use to improve pain and function in both the short and long term [7, 8]. Particularly, strength training is considered an essential part of the exercise programmes when conservative treatment is applied [9, 10]. Traditionally, quadriceps strengthening has been used for functional re-education in these patients [11]. However, currently, it is strongly recommended to combine quadriceps and proximal hip exercises in patients who suffer PFP in order to obtain better results in terms of pain reduction and improved function [6, 12].

Whole body vibration (WBV) training has demonstrated improvements in muscle strength, power, balance, flexibility, proprioception or human gait in healthy adults, the elderly [12–15]. In this type of training, vibration is transmitted from a platform on which an individual remains standing while holding on to the device with the upper limb, or even passively being the patient seated in a chair in front of the platform and the feet (barefoot) on the platform [16]. The amplitude of the vibration generated by these devices ranges from 0.7 to 14 mm, with a specific oscillation frequency that can range from 0.5 to 80 Hz [17]. Different mechanisms have been described by which vibration can contribute to flexibility improvement, especially as an acute effect, such as reduction of pain sensation, increase in blood flow or reduction of muscle stiffness [18].

In clinical settings, there is growing evidence about the positive effects of WBV training on reducing pain intensity and increasing lower limb functionality in patients with several musculoskeletal painful conditions [19, 20]. Zafar et al. [21] concluded that WBV training reduces pain and improves function in individuals with knee OA. The inclusion of the WBV in rehabilitation programs was suggested by Wang et al. [22] and Simao et al. [23] considering its benefits for improving functionality in patients with knee osteoarthritis. More recently, Wang et al. [24] supports this effect on pain reduction in patients with non-specific chronic low back pain.

To our knowledge, only one study using WBV training has been developed in patients with PFP [25]. The improvements in quadriceps and hip strength induced by the WBV make it a potential therapeutic resource for treating patients with this syndrome. The dosage of the exercise is one of the most diffuse aspects of this type of training, and it is necessary to establish optimal load parameters to maximise the benefits and reduce potential injury risks [22, 23, 26]. As far as we know, no reports exist about short-term training periods (four weeks), including knee, hip and core exercises in patients with PFP, and considering in the exercise dosage the contraction time during a global position (30 s), and not the number of repetitions of a movement. Therefore, the aim of this study was to determine the effects of adding WBV to an exercise protocol on pain and disability in patients with anterior knee pain. We hypothesised, a priori, that an exercise protocol performed on the vibratory platform for a 4-week period, with a three days per week frequency, is more effective than exercise alone for reducing pain levels and improving knee function in patients with PFP.

## Methods

### Study design

This study was a single-blind prospective randomised-controlled clinical trial, conducted between September and December 2019 at the AY360° Health and Sport Clinic (Seville, Spain).

The study was approved by the Ethical Research Committee of Jaen Province (project code 0916-N-19, approval date June 19, 2019) and prospectively registered (clinicaltrials.gov, Identifier NCT04031248). The Consolidated Standards of Reporting Trials (CONSORT) guidelines were followed. All participants provided written informed consent prior to being included in the study.

### Participants

Adults who had reported anterior knee pain were recruited by a primary care physician in a public health centre in a province of southern Spain. In order to increase the sample size of participants for the study, it was decided to expand the lower limit of the age range registered in clinicaltrials.gov, from 30 to 65 years. Based previous studies [25, 27], those participants who met the following inclusion criteria were invited to participate in the study: i) insidious onset of anterior knee pain with a duration greater than 12 weeks; ii) self-reported patellofemoral pain intensity ≥30 mm on the 100 mm Visual Analogue Scale (VAS); iii) pain provoked by at least two of the following situations: prolonged sitting or kneeling, squatting, running, hopping or ascending or descending stairs.

Participants were excluded in cases of clinical history of patellofemoral dislocation or subluxation; knee osteoarthritis (confirmed with radiological tests); knee joint effusion; concomitant injury or pain from the hip, lumbar spine, or other knee structures (meniscus, ligaments, bursa, synovial plica, infrapatellar fat); traumatic lesions of soft tissues or previous orthopaedic surgery in lower limbs; having received knee injections of corticosteroids or hyaluronic acid; cognition or impaired communication; being involved in an ongoing medical-legal dispute.

In addition, the exclusion criteria included having any contraindication for using whole body vibration, such pacemakers, arrhythmias, cardiac valve dysfunction, pregnancy, epilepsy, recent acute thrombosis, infection and recent inflammation, malignant tumours, recent implants, recent fractures, acute disc pathology, acute tendinopathy, renal lithiasis or biliary and an acute episode in rheumatic pathology.

Patients were advised not to take analgesic medication from the beginning of the study, as well as not to receive other treatments, such as physiotherapy or injection therapy. Patients in both groups were required to participate in at least 80% of the programmed sessions (10 sessions) for the analysis.

Those participants who met the inclusion criteria were randomly assigned by a member of the research team to the experimental or control group following simple randomization procedures (using a random-number generator website http://www.randomization.com, and considering a 1:1 ratio distribution of participants in the study groups).

### Study protocol

An external assistant collected all patient demographic and clinical data through interviews. A blind evaluator performed all measurements at baseline and immediately after the last treatment session for the entire sample, without knowing the group to which each participant belonged. A specific form for registering the demographic and clinical data of each participant was used. This form was filled out for each subject and codified in order to protect their privacy in accordance with the Organic Law 15/1999 on Protection of Personal Data, EU Regulation 2016/679 of the European Parliament and the Council of 27 April 2016 Data Protection as well as the Organic Law 3/2018, of December 5, on Protection of Personal Data and Guarantee of Digital Rights.

The research protocol was conducted in accordance with the Declaration of Helsinki statement of ethics, legal and regulatory principles in order to provide guidance for research related human health and all participants provided written informed consent prior to participating.

### Outcome measures

Primary outcome measure for this study was pain intensity. In addition, knee range of movement and lower limb functionality were assessed at baseline and at 4 weeks (post-treatment) using standardised instruments and cross-culturally validated patient-reported outcome measures.

#### Pain intensity and neuropathic pain

For the assessment of pain intensity during activity, VAS of 10 cm was used, where 0 corresponds to ‘no pain’ and 100 represents the ‘worst pain imaginable’ [28]. Patient were asked to express the mean intensity of his/her knee pain over time, considering for this, the last 7 days. Minimal clinically important difference for the VAS was based on a reduction of 15 mm from the baseline or a 15–20% change after intervention [29].

In addition, to assess neuropathic pain (NP), the Spanish version of the Douleur Neuropathique-4 items (DN4) was used [30]. This questionnaire has 10 items, consisting of descriptions and signs of pain that are evaluated with 1 (yes) or 0 (no), indicating patients who have a high probability of having a neuropathic pain component. The evaluations of individual items were added to obtain a maximum total score of 10 with a cut-off point ≥4.

#### Knee flexion-extension range of movement (ROM)

For the knee flexion assessment, subjects lay in a supine position with 90 degrees of hip flexion. Hip positioning was guaranteed by the use of a thigh device that aided in the maintenance of the pre-set position. A universal goniometer was placed next to the femoral lateral epicondyle. The static handle of the goniometer was aligned with the thigh, using the femoral major trochanter as a reference, while the mobile handle aligned with the leg referencing the fibula lateral malleolus [31]. For the knee extension assessment, patients lay in a supine position and the limb being evaluated was raised by the heel, with knee stabilisation in contact with the stretcher. The instrument positioning in relation to the segment was the same as in the knee flexion measurement.

#### Lower limb functional assessment

The Spanish version of the Lower Extremity Functional Scale (LEFS) was used for the lower limb functional assessment [32]. This is a short self-reported questionnaire that has been proven to be a valid and reliable tool for assessing musculoskeletal dysfunction in the lower extremity. This scale consists of 20 items, with a score of 0 to 4, where the highest score represents the highest functionality of the lower limb. It has a high correlation with the *Short Form* Health Survey (SF-36), especially with the physical function and pain subscales [26]. The minimal clinically important LEFS difference in patients with lower extremity musculoskeletal conditions is 9 points [33].

Finally, the available Spanish version of the Kujala Patellofemoral Score was filled out by participants. This 13-item questionnaire represents a specific self-report measure of knee function in patients with PFP. Seven items have a maximal score of 10 and 6 with maximal 5 points. Total score ranges from 0 to 100, where the highest scores represent a better functional capacity [34]. The Kujala score has a reported minimum clinically important difference threshold of 9.5 points in a 4-week follow-up period [35].

### Interventions

#### Whole body vibration (WBV) training

In the present study, an axial (vertical) vibration platform was used (Power-Plate® Pro 5™ AIRdaptive TM HP, Power Plate North America, Inc., Northbrook, IL, USA). This device is annually reviewed by the technical unit of the company and complies with the international Medical Devices regulations (Devices Directive [MDD] 93/42/EEC [ISO 2631. 2011, Powerplate.com, 2013]). This model has a Class IIA certificate (MDD 553319/0086), which classifies it as having a medium low risk, ensuring that the device offers a therapeutic benefit under correct use (ISO 2631:2011, Powerplate.com, 2013).

**Fig. 1:**
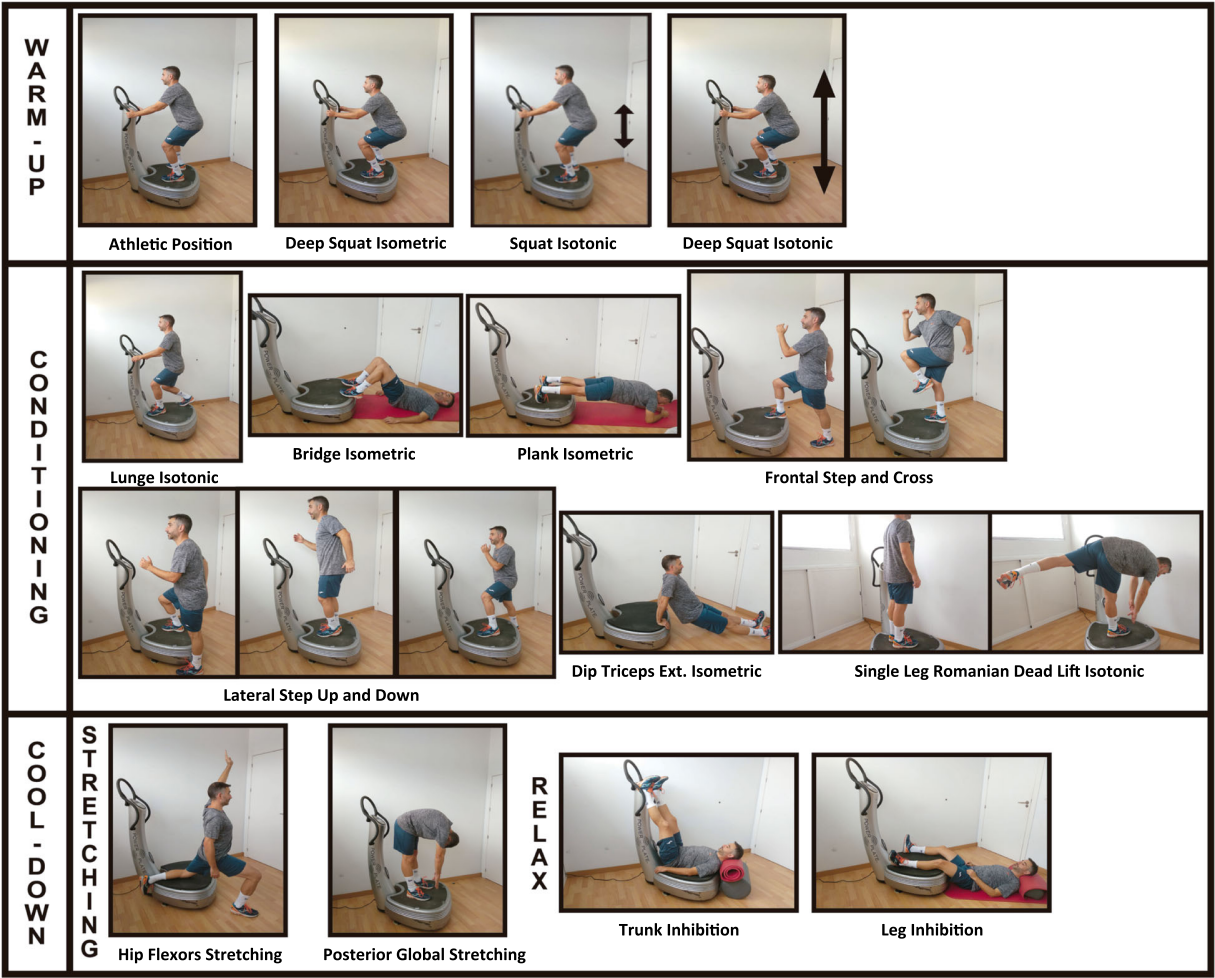
Exercise protocol of the study

The designed program consisted of a single bout of 18-exercise routine, executed on the vibration platform (Table 1). Graphic representation of the selected exercises is shown in Fig. 1.

**Table 1:** Exercise protocol of the study

PHASE		EXERCISE	WORK
WARM-UP		ATHLETIC POSITION	30 s work + 30 s rest.
		DEEP SQUAT (ISOMETRIC)	30 s work + 30 s rest.
		SQUAT (ISOTONIC)	30 s work + 30 s rest.
		DEEP SQUAT (ISOTONIC)	30 s work + 30 s rest.
CONDITIONING		LUNGE (RIGHT)	30 s work + 30 s rest.
		LUNGE (LEFT)	30 s work + 30 s rest.
		BRIDGE	30 s work + 30 s rest.
		PLANK	30 s work + 30 s rest.
		FRONTAL STEP AND CROSS RIGTH-LEFT	30 s work + 30 s rest.
		FRONTAL STEP AND CROSS LEFT-RIGHT	30 s work + 30 s rest.
		LATERAL STEP UP AND DOWN (RIGHT)	30 s work + 30 s rest.
		LATERAL STEP UP AND DOWN (LEFT)	30 s work + 30 s rest.
		DIP TRICEPS EXTENSION	30 s work + 30 s rest.
		SINGLE LEG ROMANIAN DEAD LIFT (RIGTH)	30 s work + 30 s rest.
		SINGLE LEG ROMANIAN DEAD LIFT (LEFT)	30 s work + 30 s rest.
COOL DOWN	STRETCHING	HIP FLEXORS (RIGTH)	60 s work + 6 s rest.
		HIP FLEXORS (LEFT)	60 s work + 6 s rest.
		POSTERIOR GLOBAL	60 s work + 6 s rest.
	RELAX	TRUNK INHIBITION	120 s work + 12 s rest.
		LEG INHIBITION	120 s work+ 12 s rest.

Based on the scientific literature on exercise recommendations for patients with PFP [8, 36, 37], isometric and isotonic exercises that involve core, gluteal and quadriceps muscles were selected. We also based the WBV protocol on weight-bearing position considering the recommendations for patients with anterior knee pain and due to its greater functional transfer to daily living activities [10].

The frequency of the vibration platform was fixed at 40 Hz along the study and the amplitude of the vibration platform (peak-to-peak displacement) was set at 2 mm in the first two weeks, and 4 mm during the following two [38]. The acceleration peak for these parameters were 3.2G and 6.4G respectively. In terms of force (Newtons) developed for the participants to perform the exercises, this ranged from 748.5 N in a neutral environment (without vibration, control group), to 2395.2 N (intervention group, using 40 Hz, 2 mm, 3.2G) and 4790.4 N (when parameters in the intervention group were 40 Hz, 4 mm, 6.4 G).

Each session was structured following scheduled phases of warm-up, main active part and, finally, cool-down and stretching, as recommended by the American College of Sports Medicine [39]. The warm-up phase consisted of different lower limb active exercises to increase the blood flow, muscle temperature and to activate the central nervous system [40]. All exercises in the warm-up and conditioning phases were performed considering the time on the vibration platform in sets of 30 s, with 30 s of rest between repetitions. Finally, the cool-down period involved global stretching and trunk and lower limb relaxation, with exercises involving 60 s of work and 6 s of rest and 120 s of work with 12 s of rest, respectively. The total duration of the program was 22 min, following the general lines of high-intensity aerobic interval training, which establishes a rest period that is at least equal to that of the work period [10, 41]. Based on a modified pain monitoring model, pain or discomfort only was allowed during the exercise execution if was acceptable (< 4/10) and if returned to the same baseline level of pain as before starting exercises within 24 h. If this does not occur, the exercise should be modified by reducing load (time, exercise posture or vibration amplitude) [11].

The treatment protocol comprised 12 sessions conducted over 4 consecutive weeks (3 sessions per week) and each session was supervised by an experienced clinical physiotherapist. The aim was to avoid pain during all exercises and also to avoid unusual physical activity or other additional exercises [3]. The physiotherapist assisted each participant during these sessions, supervising and correcting their positions before beginning each exercise, as well as during its execution, indicating necessary adaptations if needed. The exercise program was performed using only body weight resistance without external weight. All participants performed the exercises wearing sports shoes.

#### Control group

The control group participants were instructed to do the same supervised exercise protocol but on a vibration platform while the system was off and did not transmit any vibration to the patient’s feet.

### Statistical analysis

The statistical data processing was carried out using the PASW Advanced Statistics Package (SPSS Inc., Chicago, IL, USA), version 24.0. The data were reported as mean (standard deviation) and confidence intervals (95% CI). First, the normal distribution of the variables was verified by the Shapiro-Wilk test, following a descriptive analysis. The homogeneity of the variations was observed using the Levene test. Linearity was evaluated by bivariate scatter plots of residual values ​​observed against expected values. Comparisons between the baseline demographic and clinical data of the groups were made using the Student’s t-test for continuous data and the chi-square test for categorical data.

Separate 2 × (2) mixed-model analysis of variance (ANOVA) was used to evaluate interaction time × groups, including the time effects (baseline, post-treatment) and group effects (supervised exercise group vs WBV+ supervised exercise group) for each outcome measure. All analyses followed the intention-to-treat principle and groups were analyzed as randomized. Effect sizes were calculated using Square Eta (η^2^) was used to calculate the effect size (small, 0.01 ≤ η^2^ < 0.06, medium, 0.06 ≤ η^2^ < 0.14, and large, η2 > 0.14). The statistical significance was set at a value of *p* < 0.05.

The sample size calculation was based on the detection of: 1) a 15% change in the intensity of self-reported pain [29] and 2) a difference of > 9 points on the LEFS scale [33] and > 10 points on the Kujala scale in the comparison between groups after the intervention [35]. Taking into account the ANOVA analysis of repeated measures between factors (group x time), an alpha value of 0.05, a desired power of 90% and a medium effect size (f = 0.25), 46 participants in total were required for the study (G * Power, version 3.1.9.2).

## Results

**Fig. 2:**
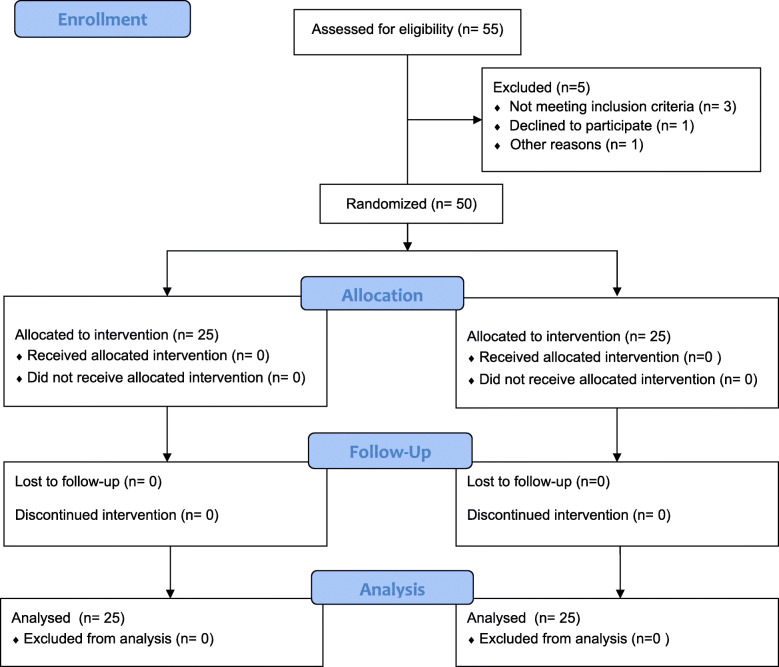
CONSORT flowchart diagram of the study participants

A sample of 55 subjects, between 19 and 67 years of age, were selected for the study. A participant flow-diagram is shown in Fig. 2. After the enrolment phase, 5 subjects were excluded due to different reasons. The final sample included 50 individuals, 24 men and 26 women (mean age ± SD, 50 ± 12.0 years).

The right lower limb side was affected for 54% of the participants (*n* = 27) and the left for 46% (*n* = 23). The participants’ level of education was: 40% primary studies (*n* = 20), 30% secondary (*n* = 15) and 30% university degree (n = 15). With respect to pharmacology treatment, 28 subjects used to take nonsteroidal anti-inflammatory drugs and analgesics before the study and 22 did not take any medication. Around 58% of the sample had an occupation in the service sector, 20% in the construction field and 22% in industry.

Table 2 shows the mean values and the standard deviation of the main variables at the baseline for both groups. The gender of the participants in each group is also presented. There were no differences between groups at the beginning of the study in any of the studied variables. Although the knee extension variable was recorded by the goniometric measurement, all the knees in the study presented a value of 0 degrees (full extension).

**Table 2:** Baseline characteristics of the participants (mean ± standard deviation)

	Total sample (*N* = 50)	WBV Group (*n* = 25)	Control Group (n = 25)	*P* Value *
Age (years)	50 ± 12.0	48 ± 13.0	52 ± 10.7	0.229
Height (cm)	167 ± 10.1	165 ± 10.7	169 ± 9.3	0.180
Weight (kg)	79 ± 16.5	76.3 ± 14.5	82.2 ± 18.1	0.205
Sex, n (%)				
*Men*	24 (48.0)	11 (44.0)	13 (52.0)	0.395
*Women*	26 (52.0)	14 (56.0)	12 (48.0)	
Body Mass Index (kg/m^2^)	28.1 ± 4.2	27.8 ± 3.8	28.5 ± 4.7	0.576
LEFS, (0–80)	50 ± 19.3	53 ± 21.3	48 ± 17.1	0.393
KUJALA, (0–100)	51 ± 19.5	50 ± 23.4	52 ± 15.1	0.700
DN4, 0–10	3.8 ± 1.85	4.2 ± 2.1	3.4 ± 1.5	0.129
VAS, mm	58 ± 17.1	56 ± 20.2	59 ± 13.6	0.581
Knee ROM_FLEX_, °	117 ± 11.7	120 ± 12.0	115 ± 10.9	0.105

Table 3 includes the differences between the baseline and post-treatment scores of the studied variables for each group and between groups. Statistical significance was found in favour of the experimental group in the between-groups comparison and in the interaction of the experimental group before and after treatment in terms of pain perception: VAS F _1, 48_ = 82.4; *p* = 0.000; η^2^ = 0.63 and DN4: F_1, 48_ = 35.7; p = 0.000; η^2^ = 0.43; as in the lower limb functionality and disability outcomes, LEFS: F _1, 48_ = 49.1; p = 0.000*;* η^2^ = 0.51 and KUJALA: F _1, 48_ = 30.5; p = 0.000; η^2^ = 0.39; and, finally, in the knee flexion range of motion: F _1, 48_ = 52.7; p = 0.000; η^2^ = 0.53.

**Table 3:** Mean differences within-group and between-groups at post-treatment in the studied variables (mean ± standard deviation or (95% confidence interval)

Group	Baseline	Post-treatment	Within-group mean changes	Between-groups mean changes
LEFS, points				
Control	48 ± 17.1	50 ± 17.0	2 (1 to 4)	20.0 (10 to 29) ^††^
WBV	53 ± 21.3	70 ± 16.0	17 (13 to 20) **	
KUJALA, points				
Control	52 ± 15.1	52 ± 13.0	0 (−3 to 3) *	19.0 (10 to 27) ^††^
WBV	50 ± 23.4	71 ± 15.6	21 (14 to 28)**	
DN4, points				
Control	3.4 ± 1.5	2.1 ± 1.2	1.3 (0.9 to 1.6) **	1.9 (1.3 to 2.4) ^††^
WBV	4.2 ± 2.1	0.2 ± .5	4 (3.1 to 4.9) **	
VAS, mm				
Control	59 ± 13.6	54 ± 14.1	5 (2 to 9)*	44.0 (35 to 52)^††^
WBV	56 ± 20.2	10 ± 15.4	46 (37 to 55)**	
KNEE ROM_FLEX,_(°)				
Control	115 ± 10.9	110 ± 13.2	5 (0 to − 7)*	23.0 (16 to 28)^††^
WBV	120 ± 12.0	133 ± 7.3	13 (9 to 15)**	
KNEE ROM_EXT_ (°)				
Control	0.4 ± 1.4	0.2 ± 1	0.2 (− 0.2 to 0.6)	0.2 (−0.5 to 0.9)
WBV	1 ± 2	0.4 ± 1.4	0.6 (− 0.08 to 1.3)	

Abbreviations: WBV, whole body vibration; LEFS-lower extremity functional scale; DN4, Douleur Neuropathique-4 items questionnaire; VAS, visual analogue scale; ROM_FLEX_, knee flexion range of motion; ROM_EXT_, knee extension range of motion

* Indicates statistically significant within-group differences (*p* < 0.05)

** Indicates statistically significant within-group differences (p < 0.001)

^††^ Indicates statistically significant between-groups differences (*p* < 0.001)

There were no adverse events reported by the participants in either group.

## Discussion

The aim of this investigation was to determine the effects of adding WBV to an exercise protocol to improve pain and disability in patients with anterior knee pain. Present findings indicate that performing knee, hip and core exercises on the vibrating platform achieves better immediate results in terms of pain relief and functional knee self-reported outcomes in comparison with exercises on their own (without a vibratory stimulus). The average change on self-reported instruments obtained by the subjects in the experimental group (VAS, LEFS and Kujala scores) reached the respective thresholds of minimal clinically important difference. Therefore, the combination of exercise and vibration may be considered to have a high short-term value in clinical practice for pain relief and improve lower limb functionality.

Pain intensity reduction after WBV training has been reported in previous studies but the mechanism for this effect remains unclear [19]. Several theories have been proposed to explain the analgesic effect of global vibration, such as the gate control or the inhibitory effect of vibration-induced non-noxious stimulus over spinothalamic tract neurons [19, 21, 23]. In addition, the stimulation generated by the vibration on the musculature through the tonic vibration reflex can generate a positive contribution to this previously mentioned analgesic effect [42]. Most likely, this marked reduction in pain intensity may be the mechanism by which patients in the experimental group showed a clinically and statistically significant improvement in self-reported knee and lower limb functionality at the end of the intervention. Avelar et al. reported that WBV may be a strategy to use to improve the functionality and self-perception of knee osteoarthritis in older adults [43]. Simao et al. also described an improvement in the self-perception of pain in elders with knee osteoarthritis after 12 weeks (3 times per week) of adding vibration to a squat exercise [23]. In our study, the intervention period is shorter but includes several exercises that can lead to the reported benefits in self-reported knee pain and functionality.

Respect to the pain quality in PFP syndrome, Jensen et al. hypothesised that the observed sensory aberrations in these patients may cause neuropathic-like knee pain [44]. Using the DN-4 questionnaire, we obtained results that could point to neuropathic components in the subjects of our sample. An alteration in the repetition or processing of A-beta inputs could generate a reduction of the pain inhibitory capacity [45]. A possible explanation for the pain reduction after WBV training may be that the increase in sensory inputs from the mechanoreceptors of the skin, joint and muscle responses caused by vibration could also favour a reduction in pain level caused by physical activity in subjects with PFP or even improve the quadriceps arthrogenic muscle inhibition [46]. In any case, better understanding of the pain mechanisms that underlying the PFP syndrome becomes essential for administering adequate treatment [44].

We suggest that the present findings about the WBV training on pain level reduction effect in short-term could help patients with PFP to overcome the negative emotional impact of this injury, which include confusion and low expectations for improvement as well as low perceived self-efficacy [3]. It is known that psychosocial impact of a musculoskeletal disease correlates with pain level and reduced physical function [47], and achieving a clinically relevant reduction in pain relief could help reduce this psychosocial impact.

In most studies, exercise programs that reported positive effects on pain intensity and muscle strength for patients with PFP were 8 weeks in duration [19]. Therefore, 4 weeks seems to be most likely a short time to show neuromuscular adaptations in these patients. In our study, we did not evaluate the effects of training on neuromuscular parameters and, therefore, we cannot confirm effects at this level. However, Abassi et al. reported beneficial effects of WBV training on the muscle strength electromyographic parameters in patients with knee osteoarthritis in a 4-week period [48]. This indicates that short-term improvements in muscle strength parameters are possible and could potentially lead to functional improvements of the self-reported scales employed. Additionally, the tonic vibration reflex, induced by the mechanical platform vibrations, can potentially increase the recruitment of the motor units and the activity of the proprioceptive system, resulting in a clinical improvement [10, 41].

In our study, a statistically significant improvement in the knee flexion range of motion after completing the protocol was observed. Osawa & Oguma, in their meta-analysis, showed that vibration interventions had significant effects on flexibility [18]. These observed changes in the knee flexion range of motion could be mediated by the knee pain level reduction. Other potential mechanism could be involved such a potential increase in blood flow after vibration or a reduction of muscle stiffness [41, 43]. In any case, we do not test directly any of these mechanisms. Nevertheless, how long the effects can be maintained after the sessions remains unclear and chronic effects of the WBV training on the knee ROM should be studied in subjects with PFP.

In this study, the exercise programme was carried out by decentralising the focus of attention on the patellofemoral joint and trying to improve the strength and function of the full lower limb and core [3, 10]. Previous investigations have shown that adding hip muscle strengthening exercises (hip abductors, external rotators and abdominal core muscles) produces a faster improvement in anterior knee pain in comparison to a standard knee rehab program [49]. Fukuda et al. reported that adding hip musculature strengthening to a knee strengthening and stretching programme in sedentary women with PFP was more effective than knee exercises alone in improving long-term function and pain outcomes. For this reason, we included additional exercises to the traditional squat training [50].

The results of this study should be considered with caution due to some methodological limitations. First, the main outcomes were obtained using self-reported measures of pain and knee functionality without any other neuromuscular objective parameters. A longer follow-up period is most likely needed in order to objectively register neuromuscular adaptations. Second, only acute effects on clinical outcomes were assessed during the post-intervention period and, therefore, we do not know how long the registered benefits can be maintained. Studies with longer follow-up periods are necessary. Third, we did not stratify our sample into subgroups of patients with homogeneous clinical statuses as has been suggested [5]. Due to the complex nature and aetiology of this painful syndrome, it is necessary to group patients with similar clinical characteristics together in order to know the effects of clinical interventions more specifically. For example, in this work, although not statistically different there are some differences in the subject’s characteristics such age or weight (i.e. intervention group subjects are a little younger than controls) which could have influenced the results. Finally, potential adaptations on the trained musculature have not been evidenced. To the best of our knowledge, previous results of WBV training have been reported in women with PFP [51]. We have reported results for a male sample, which should be contrasted in future studies with a greater sample and a longer follow-up period.

## Conclusion

In conclusion, the realisation of a training protocol of hip, knee and core exercises on a vibratory platform produces positive effects on the pain level and functional capacity of patients with PFP and is more effective than exercise alone in improving pain and function in patients with PFP in the short term.

## Data Availability

The datasets analysed in the current study are available from the corresponding author on reasonable request.

## Abbreviations

WBV: Whole body vibration
PFP: Patellofemoral pain
LEFS: Lower extremity functional scale
VAS: Visual Analogue Scale
NP: Neuropathic pain
DN4: Douleur Neuropathique 4-item scale
SD: Standard deviation
CI: Confidence interval
ANOVA: Analysis of variance
